# 63-Year-Old Male with Gastric Outlet Obstruction

**DOI:** 10.1155/2014/767165

**Published:** 2014-09-15

**Authors:** Bhavraj Khalsa, Patrick Rudersdorf, Dattesh Dave, Brian R. Smith, Chandana Lall

**Affiliations:** ^1^Department of Radiology, University of California, Irvine Medical Center, Orange, CA 92868, USA; ^2^Department of Surgery, University of California, Irvine Medical Center, Orange, CA 92868, USA; ^3^University of California, Irvine School of Medicine, Irvine, CA 92697, USA; ^4^Department of Surgery, Long Beach Veterans Affairs Medical Center, Long Beach, CA 90822, USA

## Abstract

We describe a case of a 63-year-old male with complicated Bouveret's syndrome, both in its presentation and in its management. Bouveret's syndrome is a rare cause of gastric outlet obstruction resulting from mechanical obstruction from gallstones at the pyloroduodenal segment. As Bouveret's syndrome can be a diagnostic and therapeutic challenge for clinicians, we aim to identify clinical and radiologic pearls that can lower the threshold for the diagnosis of Bouveret's syndrome.

## 1. Introduction

The French physician Leon Bouveret first described two cases of gastric outlet obstruction caused by gallstones in 1896. Obstruction at the gastric outlet is most commonly attributed to chronic ulcers and subsequent stenosis of the pylorus or malignancy at the prepyloric antrum [1]. Gallstones causing small bowel obstruction are a rare occurrence and are more commonly attributed to gallstone ileus, which refers to a mechanical obstruction of the small bowel by a gallstone at the ileocecal junction and occurs via fistulization of the gallbladder into the small bowel [2]. Obstruction from gallstones much more proximally, at the pyloroduodenal segment, is a much rarer variation of an already rare phenomenon. A Pubmed search for “Bouveret's syndrome” reveals largely case reports and very few review papers, owing to its rare presentation and challenging diagnosis. We describe a case of complicated Bouveret's syndrome, both in its presentation and in its management. We also aim to identify clinical and radiologic pearls that can lower the threshold for the diagnosis of Bouveret's syndrome.

## 2. Case Report

A 63-year-old male with multiple medical problems was referred to the general surgery service for surgical evaluation for high-grade gastric outlet obstruction seen on CT scan. The patient presented with intolerance to oral intake, three days of worsening nausea and nonbloody/nonbilious vomiting, throat fullness, and a feeling of a “brick” in his stomach. He also complained of a ten-pound weight loss over the previous three months. He reported his last bowel movement to be four days prior to presentation and flatus one day prior to presentation.

The patient's past medical history was significant for gastroesophageal reflux disease (GERD), insulin-dependent diabetes mellitus, hyperlipidemia, nonischemic cardiomyopathy (ejection fraction of 20%), and coronary artery disease status-post STEMI and stent placement (five years prior to presentation). The patient also had two recent admissions to the medicine service within the previous three months for progressively worsening atypical chest pain (most recently 1 month prior to presentation). Due to the patient's significant cardiac history, he was evaluated and ruled-out for acute coronary syndrome during both admissions and discharged with a diagnosis of GERD on a high dose H2-blocker and proton-pump-inhibitor.

He was also referred to the gastroenterology service for further management of his GERD, and it was felt at this time that he may benefit from reflux surgery. However, given the patient's cardiac history, it was decided to continue with maximal medical therapy and to consider reflux surgery if his symptoms did not improve in three-month time. The patient's history was also notable for an episode of gallstone pancreatitis (four years prior to presentation). Of note, during this episode, he underwent an ERCP with needle knife sphincterotomy due to inability to cannulate the common bile duct (CBD) with a sphincterotome. A cholangiogram at that time showed a dilated CBD and intrahepatic ducts with a filling defect within the CBD, with balloon sweeps showing debris but no definitive stones.

**Figure 1:**
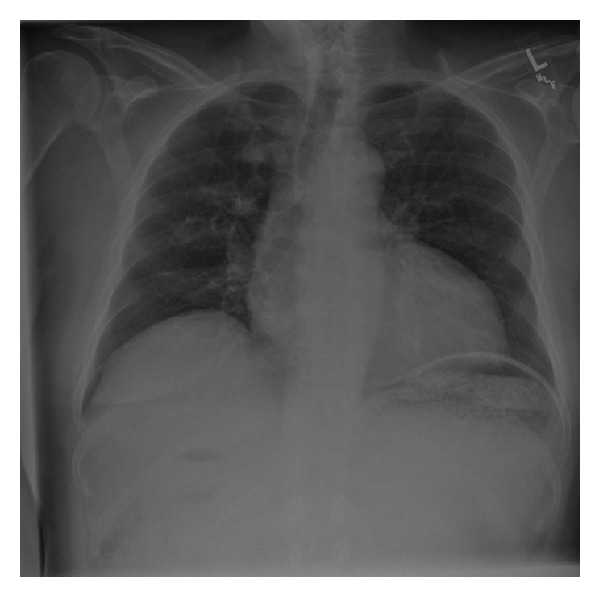
Chest X-ray of patient demonstrating enlarged gastric silhouette secondary to high-grade gastric outlet obstruction that could be mistaken for free air under the diaphragm.

**Figure 2:**
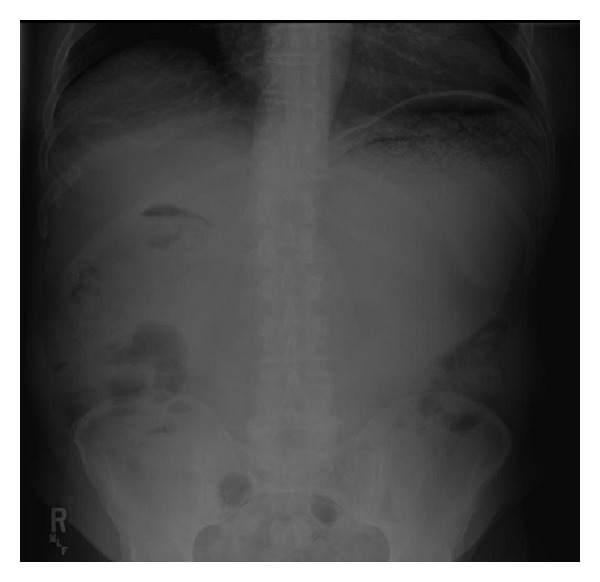
Abdominal X-ray showing dilated and partially fluid-filled stomach.

**Figure 3:**
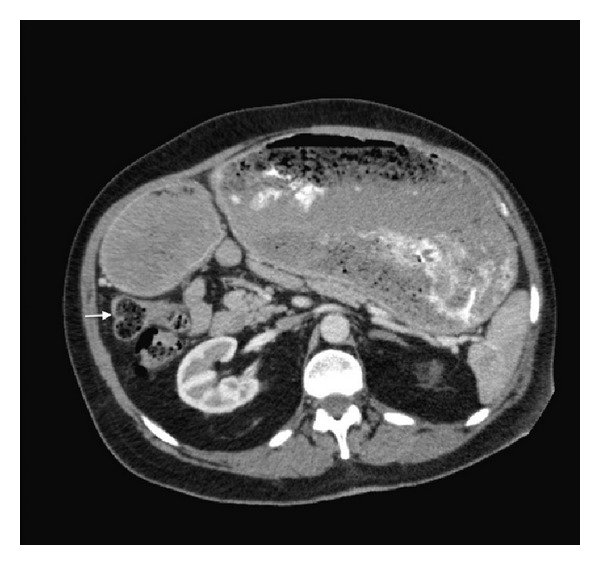
Coronal CT showing marked gastric distension with large amount of intraluminal gastric contents. A small bowel feces sign (arrow) is also appreciable in the region of the pylorus and the first portion of the proximal duodenum with surrounding adjacent inflammatory changes and mucosal wall thickening.

**Figure 4:**
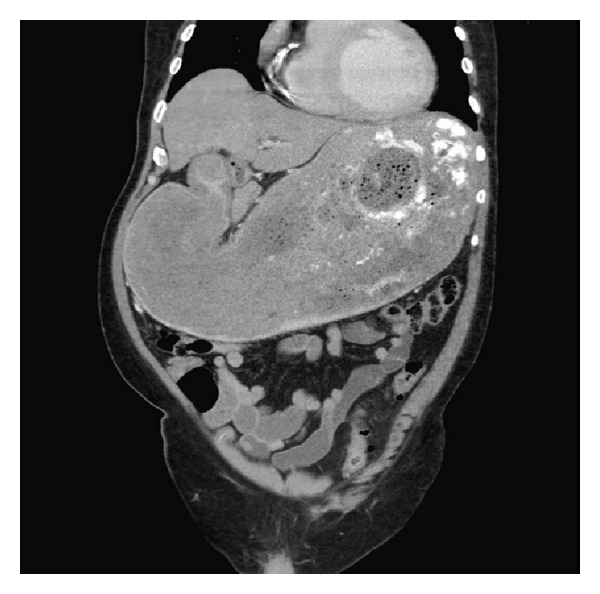
Sagittal view showing massive gastric distension with no passage of oral contrast beyond the gastric fundus. The visualized loops of bowel are decompressed.

**Figure 5:**
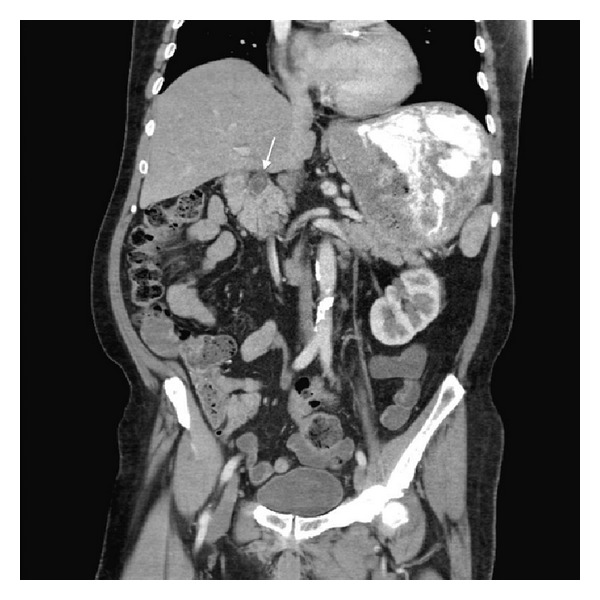
Sagittal view showing 1.6 cm common bile duct stone (arrow) with associated 1.6 cm common bile duct dilatation and minimal intrahepatic biliary ductal dilatation. Again, oral contrast only in the region of the gastric fundus, decompressed loops of bowel, and a small bowel feces sign in the region of the pylorus and the proximal duodenum are seen.

Physical exam revealed dry mucous membranes, moderate abdominal distention, epigastric fullness, and hypoactive bowel sounds. Although there was abdominal discomfort, frank tenderness was absent. The patient's laboratory values were also unremarkable, with normal complete blood count, basic metabolic panel, liver function tests (except for an albumin of 3.1), and amylase and lipase. Chest X-ray (Figure 1) showed enlargement of the gastric silhouette and abdominal X-ray (Figure 2) showed a dilated and partially fluid filled stomach. A CT of the abdomen and pelvis with oral and IV contrast was then ordered to further evaluate the patient's gastric distension. The study revealed marked gastric distension with large amount of intraluminal gastric contents/bezoar (Figures 3 and 4) and a small bowel feces sign in the region of the pylorus and first portion of the proximal duodenum with surrounding inflammatory changes and mucosal wall thickening. There was no passage of oral contrast beyond the stomach. Additionally, a partially calcified 1.6 cm stone within the CBD (Figure 5) with associated 1.6 cm CBD dilation and minimal intrahepatic biliary ductal dilation was also visualized.

As a result of these findings, the patient was taken to the operating room for an esophagogastroduodenoscopy (EGD) for aspiration of the gastric contents and biopsies of any potentially obstructing masses. Due to the significant amount of gastric fluid and bezoar encountered, however, visualization proved difficult resulting in a prolonged and tedious aspiration of gastric contents. Eventually, after aspiration of approximately five liters of fluid, a pinpoint pylorus and a stiff, nonpliable, and nondistensible gastric antrum was visualized. The significantly narrowed pylorus could not be entered despite attempts with a pediatric endoscope and balloon dilation. As such the scope was removed after several biopsies of the pylorus and antrum were taken due to concern for carcinoma as the underlying etiology for the patient's gastric outlet obstruction given no obvious findings of ulcers and the stiffened pylorus and antrum. A nasogastric tube was placed for continued gastric decompression.

The biopsy results returned negative for dysplasia, malignancy, or* Helicobacter pylori*. Due to the uncertain etiology of the patient's condition as well as the preoperative finding of choledocholithiasis on abdominal CT, the patient was taken to the operating room after five days of nasogastric decompression for a diagnostic laparoscopy and possible exploratory laparotomy. The diagnostic laparoscopy revealed no evidence of metastases and the procedure was converted to an exploratory laparotomy. Deeper dissection again revealed no evidence of malignancy but, however, did reveal a markedly atrophic gallbladder and a fistula between the body and the first portion and the duodenum. Once the fistula was taken down, numerous gallstones were expressed from within the duodenum at the cholecystoduodenal fistula site. Following removal of the gallstones a cholangiogram was performed and a filling defect was noted in the distal CBD. A balloon Fogarty catheter was used to express a large common bile duct stone and a repeat cholangiogram no longer revealed a filling defect. A T-tube was inserted and a retrogastric-retrocolic gastrojejunostomy was then performed to bypass the patient's high-grade gastric outlet obstruction.

The patient's postoperative hospital course was complicated by episodes of fever, hypotension, and slow return to bowel function. He was discharged home on postoperative day thirteen after successfully tolerating an oral diet. On subsequent clinical follow-up visits, the patient was recovering well from his surgery and had regained some of his weight.

## 3. Discussion

Bouveret's syndrome is an uncommon cause of gallstone ileus with a proximal gastrointestinal obstruction in comparison to the relatively much more common distal small bowel obstruction found in gallstone ileus. It is characterized by an obstruction at the level of the gastroduodenum resulting in upper gastrointestinal obstructive symptoms from gastric outlet obstruction. A recent review of 128 patients with Bouveret's syndrome provides a listing of common clinical characteristics in order to aid in diagnosis [3]. The clinical diagnosis, however, is a challenging one to make because of the nonspecific nature of presentation and rare incidence. As such there are no diagnostic criteria or clinical triads for the diagnosis of Bouveret's syndrome. Even though our patient met a number of the listed features (nausea/vomiting, abdominal discomfort, recent weight loss, anorexia, constipation, signs of dehydration, abdominal distension, and hypoactive bowel sounds), none of these findings are pathognomonic for the syndrome and necessitated consideration of other causes including the more common infectious and malignant etiologies.

In addition to clinical features, the review also provides findings on several imaging modalities that lower the threshold for prompt intervention. CT findings include Rigler's triad for gallstone ileus (pneumobilia, mechanical bowel obstruction, and an ectopic gallstone), which was present in approximately 77% of patients with gallstone ileus [4]. In comparison, pneumobilia was present in only 60%, ectopic gallstones were in only 42%, and evidence of gastric or duodenal obstruction was in only 33% of patients with Bouveret's syndrome [3]. As such, further efforts have been made to characterize the CT findings of Bouveret's syndrome in order to achieve a higher accuracy in diagnosis [5]. These additional subtle findings include crescents of intraluminal compressed dependent air, faint radiolucency outside a calcific rim, focal dilatation of the small bowel, and soft tissue density around a calcified ectopic stone. An increase in sensitivity with this approach, however, is not reported. In retrospect our case included none of these signs.

Our experience revealed findings on diagnostic workup that were particularly unrevealing in the setting of high-grade gastric outlet obstruction. An abdominal film revealed massive gastric distension, while computed tomography revealed a massively enlarged stomach filled with gastric contents, without an obvious source of the patient's gastric outlet obstruction. A common bile duct stone was visualized; however, there was no associated biliary ductal dilatation beyond the size of the stone, likely due to decompression from the cholecystoduodenal fistula found intraoperatively. In addition, the patient's liver function tests were within normal limits, also supporting biliary decompression via a fistula in the setting of a likely long-term biliary obstruction. Further raising the suspicion for Bouveret's syndrome was the unusual location for a “small bowel feces sign” (Figures 3 and 5) that was evident at the level of the pylorus and the first portion of the proximal duodenum. The radiologic sign is most often seen at the distal small intestine at a location immediately proximal to the site of obstruction and is caused by the slow transit time that allows for increased water absorption and the formation of feces-like intestinal content [6]. Finally, the CT did not reveal any focal gastric or pancreatic lesions that would explain the patient's high-grade obstruction. As such, in our case, the patient's diagnosis was suspected preoperatively but, however, was confirmed and managed intraoperatively.

In order to confirm our suspicion, we first attempted a minimally invasive endoscopic approach due to the patient's multiple comorbidities and a reasonable rate of diagnostic success [3]. Several treatment options for Bouveret's syndrome have been previously described, and endoscopic stone retrieval is now the preferred modality, especially for patients with multiple comorbidities [7]. In addition to endoscopic retrieval, other minimally invasive techniques include lithotripsy (laser, mechanical, electrohydraulic, or extracorporeal shock wave) and argon plasma coagulation [8]. Despite multiple attempts, however, EGD was nondiagnostic in this setting as we were unable to pass the pinpoint pylorus. In this setting of complicated Bouveret's syndrome, open surgery remains the treatment option of choice [9]. Surgical options include laparotomy, longitudinal duodenotomy, stone retrieval, and transverse closure of duodenotomy. Depending on the intraoperative findings, other options include gastrostomy, enterolithotomy, cholecystectomy with or without fistula repair, laparoscopic enterolithotomy with or without fistula repair, and pyloroplasty. Due to the extensive degree of obstruction in our patient, our surgical intervention also included a gastrojejunostomy to bypass the patient's proximal obstruction. As such, the specific treatment approach depends on multiple factors, including the preoperative clinical features, the information contained within diagnostic imaging workup, and the associated complicated features that preclude certain treatment approaches.

Bouveret's syndrome can be a diagnostic and therapeutic challenge for clinicians and there are multiple features that should lower the threshold for diagnostic consideration. In our experience, these features include gastric outlet obstruction in the absence of ulcers, infection (*Helicobacter pylori*), or malignancy and in the presence of suspicious CT findings that include a large impacted common bile duct stone with a decompressed biliary system and a small-bowel feces sign in the early proximal small bowel. Still, none of these findings are pathognomonic for the condition and the treatment approach should take into account the patient's associated comorbidities and associated complicated features.
